# 

**Published:** 1938

**Authors:** 


### CONTENTS.

| | Page |
|---|---|
| **Some Problems in the Diagnosis and Treatment of Intra-Cranial Tumours.** By F. Wilfred Willway, M.D., M.S., B.Sc., F.R.C.S. | 151 |
| **Some Tuberculous Conditions.** By Hubert Chitty, M.S., F.R.C.S. | 161 |
| **The Vitamin B Complex.** By H. M. Sinclair, M.A., B.Sc., B.M., B.Ch. | 173 |
| **The Medicinal Waters at Burnham-on-Sea.** By J. A. Nixon, C.M.G., M.D., F.R.C.P. | 189 |
| **Reviews of Books** | 199 |
| **Editorial Notes** | 203 |
| **Obituary:** Cyril George Russ Wood, O.B.E., F.R.C.S. | 205 |
| **The Medical Library of the University of Bristol** | 207 |
| **Publications Received** | 209 |
| **Local Medical Notes** | 210 |

